# Remodeling of B-Cell Subsets in Blood during Pegylated IFNα-2a Therapy in Patients with Chronic Hepatitis B Infection

**DOI:** 10.1371/journal.pone.0156200

**Published:** 2016-06-09

**Authors:** Caroline Aspord, Juliana Bruder Costa, Marie-Christine Jacob, Tania Dufeu-Duchesne, Inga Bertucci, Noelle Pouget, Ophelie Brevot-Lutton, Fabien Zoulim, Marc Bourliere, Joel Plumas, Vincent Leroy, ANRS HB06 PEGAN study group

**Affiliations:** 1 University Joseph Fourier, Grenoble, F-38041, France; INSERM, U823, Immunobiology & Immunotherapy of Cancers, La Tronche, F-38706, France; 2 EFS Rhone-Alpes, R&D Laboratory, La Tronche, F-38701, France; 3 CHU Grenoble, Michallon Hospital, Hepato-gastroenterology unit, Grenoble, F-38043, France; 4 University Joseph Fourier, Grenoble, F-38041, France; INSERM, U823, CRI/Institut Albert Bonniot, Grenoble, F-38000, France; Department of Immunology, CHU de Grenoble, Grenoble, F-38000, France; 5 University Joseph Fourier, Grenoble, F-38041, France; INSERM, U823, Analytic Immunology of chronic pathologies, La Tronche, F-38706, France; 6 ANRS (France REcherche Nord & sud Sida-hiv Hépatites: FRENSH), Paris, France; 7 Sorbonne Universités, UPMC Univ Paris 06, INSERM, Institut Pierre Louis d’épidémiologie et de Santé Publique (IPLESP UMRS 1136), 75012, Paris, France; 8 INSERM U1052—CNRS 5286, Cancer Research Center of Lyon (CRCL), Lyon, France; 9 Hepatology Department, Hospices Civils de Lyon, Lyon, France; 10 Université de Lyon, Lyon, France; 11 Hepato-gastroenterology department, Hospital Saint Joseph, Marseille, 13008, France; CRCL-INSERM, FRANCE

## Abstract

The ultimate goal of pegylated interferon-alfa-2a (Peg-IFN-α) therapy in chronic hepatitis B (CHB) infection is HBsAg seroconversion. Even though B cells are major mediators of a positive clinical outcome, their modulation during Peg-IFN-α therapy has not yet been described. We investigated here the effects of Peg-IFN-α on eight circulating B-cell subsets thanks to an original multi-gating approach based on CD19, CD27, IgD, CD10, and CD38 markers in patients with CHB treated with nucleos(t)ide analog alone or in combination with Peg-IFN-α. These dynamic changes were analyzed during the 48-weeks of Peg-IFN-α therapy and up to 2 years after the cessation of treatment. The CD19^+^CD27^-^IgD^+^CD10^+^CD38^high^ transitional B cells and the CD19^+^CD27^+^IgD^-^CD10^-^CD38^high^ plasmablasts continuously increased, whereas the CD19^+^CD27^-^IgD^+^CD10^-^CD38^low^ naive, CD19^+^CD27^+^IgD^+^ natural memory, and CD19^+^CD27^+^IgD^-^CD10^-^CD38^low^ post-germinal center B cells decreased during the course of Peg-IFNα treatment. Such modulations correlated with a sustained increase in sCD30 levels and the decrease in plasma HBsAg. However, no seroconversion occurred and all parameters returned to baseline after the stop of the treatment. Peg-IFN-α therapy mediates a remodeling of B-cell compartmentalization, without clinical relevance. Our study provides new insights into the immunomodulatory effects of Peg-IFN-α on circulating B-cells, and questioned the benefit of the add-on Peg-IFN-α treatment in CHB.

## Introduction

In its pegylated form, interferon-alpha-2a (Peg-IFN-α) was used in the treatment of chronic HBV, because it possesses strong antiviral and immunomodulatory properties stimulating both innate and adaptive immune responses. Recently, Peg-IFN-α has been considered as a therapeutic alternative to the prolonged use of nucleos(t)ide analogs (NA) in chronic HBV (CHB) infection [1–3], due to its potential to trigger a sustained virological response off-treatment and HBsAg seroconversion [4]. In this context, B-cell responses appear to be critical in the control of infection. Although recent clinical trials described the impact of Peg-IFN-α on the major anti-viral immune effectors such as T cells and NK cells [5–8], nothing is known regarding the modulation of B cells in CHB patients treated with Peg-IFN-α.

In the context of HBV, B-cell responses are a T-cell-dependent process and lead to an efficient antibody production in patients who manage to clear the virus. The anti-HBV antibodies exert viral clearance through the formation of complexes with free viral particles removing them from circulation or preventing their attachment and uptake by hepatocytes [9]. HBV-specific antibodies are indicators of specific stages of the disease. Whereas HBsAg-specific antibodies are neutralizing and mediate protective immunity, HBcAg-specific and HBeAg-specific antibodies persist for life after clinical recovery [10]. These specific antibodies are usually undetectable in patients with CHB infection. In addition to their essential role in humoral immunity, B cells are also involved in capturing and concentrating antigens for presentation, in producing immunomodulatory cytokines, in influencing T-cell and DC responses, and in initiating subsequent T-cell immune responses. They contribute towards distinct functions during the immune response in vivo, and affect lymphoid tissue structures [11, 12].

Current schemes of classification of human B-cell populations found in the secondary lymphoid tissue and in peripheral blood are based on the expression of six major surface markers: CD10, CD19, IgD, IgM, CD38, and CD27 that provide the identification of different stages of mature B-cell development and on the description of the B-cell subsets: transitional B cells, naive B cells, natural effector memory B cells, pre-germinal center (GC) B cells, GC B cells, memory B cells, and plasmablasts [13, 14].

We designed a unique and original strategy of classification based on these markers to investigate for the first time the impact of Peg-IFNα therapy on eight peripheral B-cell subsets, on B cell-modulating soluble factors, such as BAFF/APRIL, sCD26, and sCD30, and on the levels of IgG and IgM immunoglobulins in patients with CHB. We compared HBeAg-negative patients treated with NA alone and patients receiving NA in combination with a 48-week course of Peg-IFN-α, before treatment, at different time points during the course of Peg-IFN-α therapy, and up to 2 years (week (W)144) after the end of the treatment. Our results revealed a major impact of Peg-IFN-α therapy on peripheral B-cell subsets and a complete remodelling of the B-cell compartment. This study provides new insight into the immunomodulatory effect of Peg-IFN-α, but also reveals the absence of prognostic relevance, which questioned the benefit of the add-on Peg-IFN-α treatment over the NA or Peg-IFN-α monotherapies.

## Materials and Methods

### Patients

The study participants comprised 23 HBsAg-positive and HBeAg-negative patients with CHB treated by analogs who had undetectable HBV-DNA for at least one year and were enrolled in a multicenter, randomized, phase 3 study of Peg-IFN-α (ANRS HB06 PEGAN, registered as NCT01172392). Fourteen patients remained on nucleos(t)ide analogs alone (control group) whereas nine received an additional 180 μg Peg-IFN-α (Pegasys; F Hoffmann-La Roche, Basel, Switzerland) once a week for 48 weeks (IFN group). All participants signed informed consent forms. The study protocol was conducted according to the Declaration of Helsinki and French law for biomedical research. It was approved by the Ethics Committee CPP Sud Méditerranée I and the French Regulatory Authority (ANSM). The main features of the patients are shown in Table 1. Heparinized peripheral blood samples were obtained at baseline and after 4 (Peg-IFN-α only), 12, 24, 48, 96, and 144 weeks of treatment. Peripheral blood mononuclear cells (PBMCs) were purified by Ficoll-Hypaque density gradient centrifugation (Eurobio) and the total lymphocyte concentration was determined. Plasma samples were collected before and at each time point of treatment and stored frozen at -80°C.

**Table 1:** Clinical features of patients at baseline and during the course of the treatment.

					Baseline					HBsAg (log IU/ml)						Anti-HBs Ab status			
Case #	Group	Age (year)	Sex	Analog	HBV DNA (IU/ml)	AST (IU/ml)	ALT (IU/ml)	Metavir activity	Metavir fibrosis	W0	W12	W24	W48	W96	W144	W0	W48	W96	W144
1	**NA**	55	M	tenofovir	<20	22	21	2	4	2,85	2,97	2,93	2,89	2,83	ND	neg	neg	neg	neg
2		54	M	entecavir	<20	27	24	1	1	3,17	3,15	3,16	3,02	2,82	ND	neg	neg	neg	neg
3		56	M	tenofovir	<20	38	54	ND	4	1,72	1,72	1,72	1,65	1,60	ND	neg	neg	neg	neg
4		34	M	entecavir	<20	27	51	1	0	3,71	3,74	3,51	3,54	3,49	ND	neg	ND	neg	ND
5		31	M	tenofovir	<20	28	28	1	0	4,07	3,97	3,99	4,13	3,98	ND	neg	neg	neg	ND
6		51	M	tenofovir	<20	22	35	ND	ND	3,08	2,99	3,04	3,05	3,05	2,80	neg	ND	neg	neg
7		39	M	entecavir	<20	19	51	3	3	3,11	3,08	3,13	3,11	3,09	2,92	neg	neg	neg	neg
8		54	M	tenofovir	<20	23	29	2	2	3,41	3,32	3,34	3,16	3,08	ND	neg	ND	neg	neg
9		35	M	tenofovir	<20	35	38	2	4	3,39	3,28	3,36	3,19	3,12	ND	neg	neg	neg	ND
10		48	F	lamivudine	<20	19	15	3	1	2,60	2,55	2,38	2,56	2,51	2,24	neg	neg	neg	neg
11		65	M	tenofovir	<20	30	25	1	4	0,61	0,61	0,18	0,18	-0,52	ND	neg	neg	neg	pos
12		37	M	entecavir	<20	43	35	2	1	4,32	4,30	4,21	4,14	4,42	ND	neg	neg	neg	ND
13		62	F	lamivudine	<20	23	19	1	2	3,36	ND	3,40	3,22	3,35	ND	neg	neg	neg	neg
14		35	M	tenofovir	<20	24	24	2	4	3,51	3,44	3,50	3,57	3,47	3,54	neg	neg	neg	ND
15	**NA +** Peg-IFN-α	41	M	entecavir	<20	28	39	3	2	3,66	3,55	3,53	3,00	3,53	3,37	neg	neg	neg	neg
16		44	M	entecavir	<20	23	41	1	2	3,35	3,21	3,24	3,10	3,36	ND	neg	neg	neg	neg
17		63	M	tenofovir	<20	31	42	1	1	2,93	2,97	2,83	2,59	2,65	ND	neg	neg	neg	ND
18		57	M	entecavir	<20	19	28	2	4	2,88	2,89	2,79	2,67	2,61	2,34	neg	neg	neg	neg
19		49	M	entecavir	<20	32	20	ND	ND	3,90	3,78	2,70	1,28	3,26	2,90	neg	neg	neg	neg
20^a^		49	F	tenofovir	<20	17	13	1	2	2,38	2,24	2,36	2,20	1,61	ND	neg	neg	neg	ND
21		67	M	tenofovir	<20	40	56	2	2	3,78	3,64	3,24	1,83	3,08	3,06	neg	neg	neg	neg
22		34	M	entecavir	<20	24	22	ND	ND	4,36	4,27	4,27	4,34	4,35	ND	neg	neg	neg	ND
23		61	M	adefovir	<20	25	25	2	4	2,89	2,84	2,31	2,21	1,86	ND	neg	neg	ND	ND

a: patient stopped treatment after W12

NA: nucleos(t)ide analog; ND: not determined

### Phenotypic analysis

**Fig 1:**
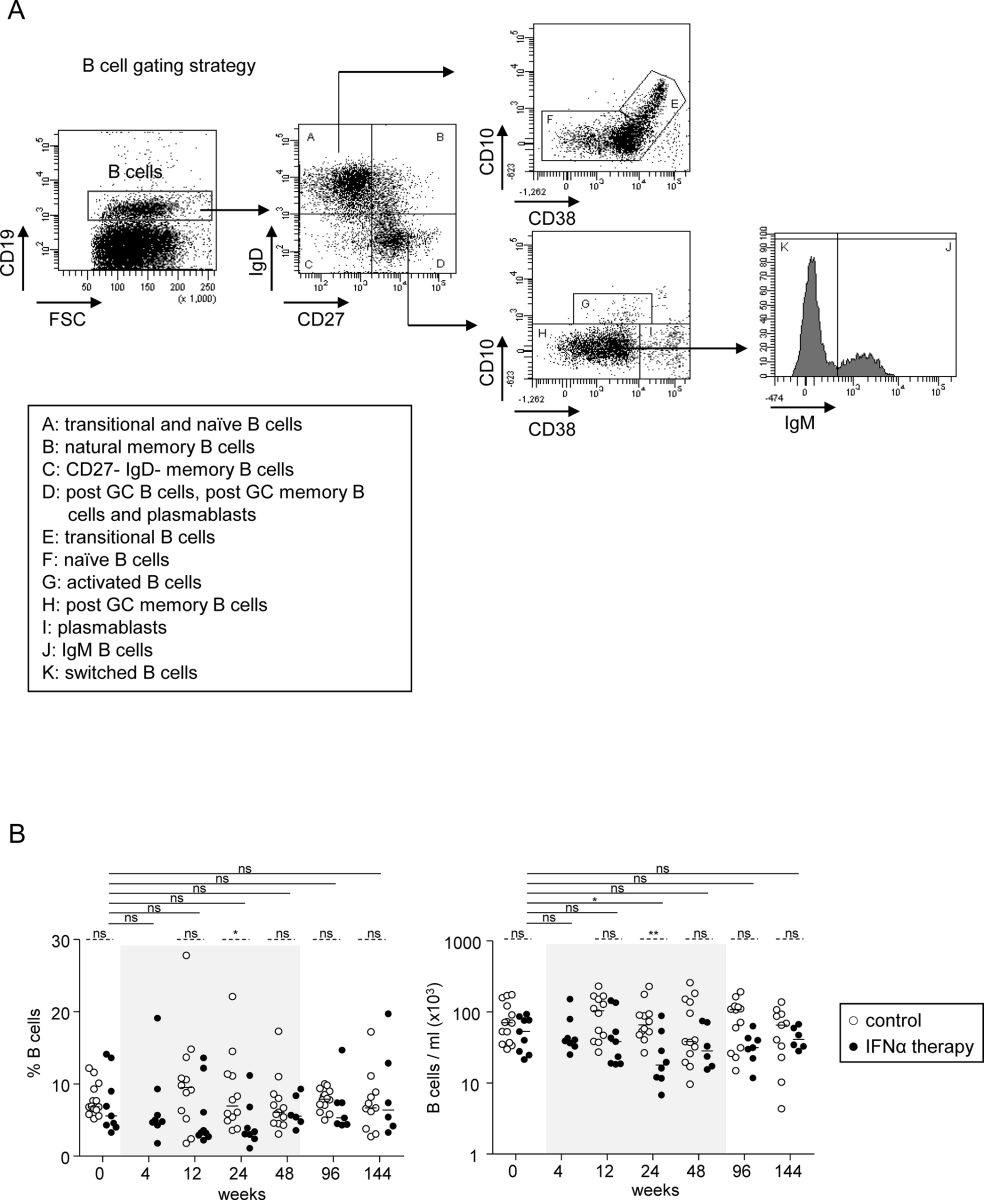
B-cell subsets during Peg-IFN-α therapy. (A) B-cell gating strategy. The peripheral B-cell subsets were classified according to the most common lineage/differentiation markers CD19, CD10, CD27, CD38, IgD, and IgM. B-cell subsets were defined as: transitional B cells (CD19^+^CD27^-^IgD^+^CD10^+^CD38^high^); naive B cells (CD19^+^CD27^-^IgD^+^CD10^-^CD38^low^); natural memory B cells (CD19^+^CD27^+^IgD^+^); post-GC memory B cells (CD19^+^CD27^+^IgD^-^CD10^-^CD38^low^); plasmablasts (CD19^+^CD27^+^IgD^-^CD10^-^ CD38^high^); CD27^-^IgD^-^ memory B cells (CD19^+^CD27^-^IgD^-^) and activated B cells (CD19^+^CD27^+^IgD^-^CD10^+^CD38^low^). Post-GC memory B cells were further subdivided into IgM^+^ and IgM^-^ switched B cells. Representative dotplots from one patient with CHB infection. (B) Modulation of total B cells by Peg-IFN-α. Frequency (within PBMC) and absolute numbers of total B cells in patients with CHB infection treated with nucleos(t)ide analog alone (*open circles*, n = 11–14) or together with Peg-IFN-α (*black circles*, n = 8–9). The gray area represents the period of Peg-IFN-α administration. Bars represent median. *P* values were calculated using the Wilcoxon test (*straight lines*) or the Mann-Whitney test (*dashed lines*). * p<0.05, ** p<0.01, *** p<0.001.

To analyze the B-cell subsets, PBMCs were stained with fluorochrome-labelled anti-human CD10-APC, CD19-APC-Cy7, CD27-PC7, CD38-PercCP-Cy5.5, IgD-FITC, and IgM-PE (BD Biosciences, San Jose, CA, USA). Antibody-stained cells were analyzed by flow cytometry using FACSCANTOII and Diva software (BD Biosciences). The gating strategy is outlined in Fig 1A. The absolute numbers of subsets were obtained by multiplying their percentage by the total lymphocyte number.

### Soluble factor and immunoglobulin measurements

Levels of BAFF, APRIL, soluble CD26, and soluble CD30 proteins were determined in plasma by specific ELISA kits according to manufacturer’s instructions (eBioscience). Levels of IgG and IgM immunoglobulins were quantified by a Cytometric Bead Array assay (BD Biosciences).

### Statistical analysis

Statistical analysis was performed using Mann-Whitney non-parametric U-test, Wilcoxon matched pairs test, and Spearman correlation using Prism software.

## Results

### Modulation of B-cell compartment by Peg-IFN-α

We investigated the immunomodulatory effects of Peg-IFN-α on B cells by first analyzing the frequencies and absolute numbers of total B cells before treatment and at different time points during treatment (Fig 1A). Patients were compared according to their treatment arm (Table 1). In response to Peg-IFN-α, we observed a progressive decrease in the frequencies and absolute numbers (Fig 1B) of total B cells (CD19+), which troughed at W24 compared to baseline level, going from 7.3% to 4.4% and from 55.7x10^3^/ml to 29.7x10^3^/ml respectively. These parameters were restored at the end of Peg-IFN-α treatment.

### Peg-IFN-α modulates B-cell subset distribution without affecting HBV-specific immunoglobulins

**Fig 2:**
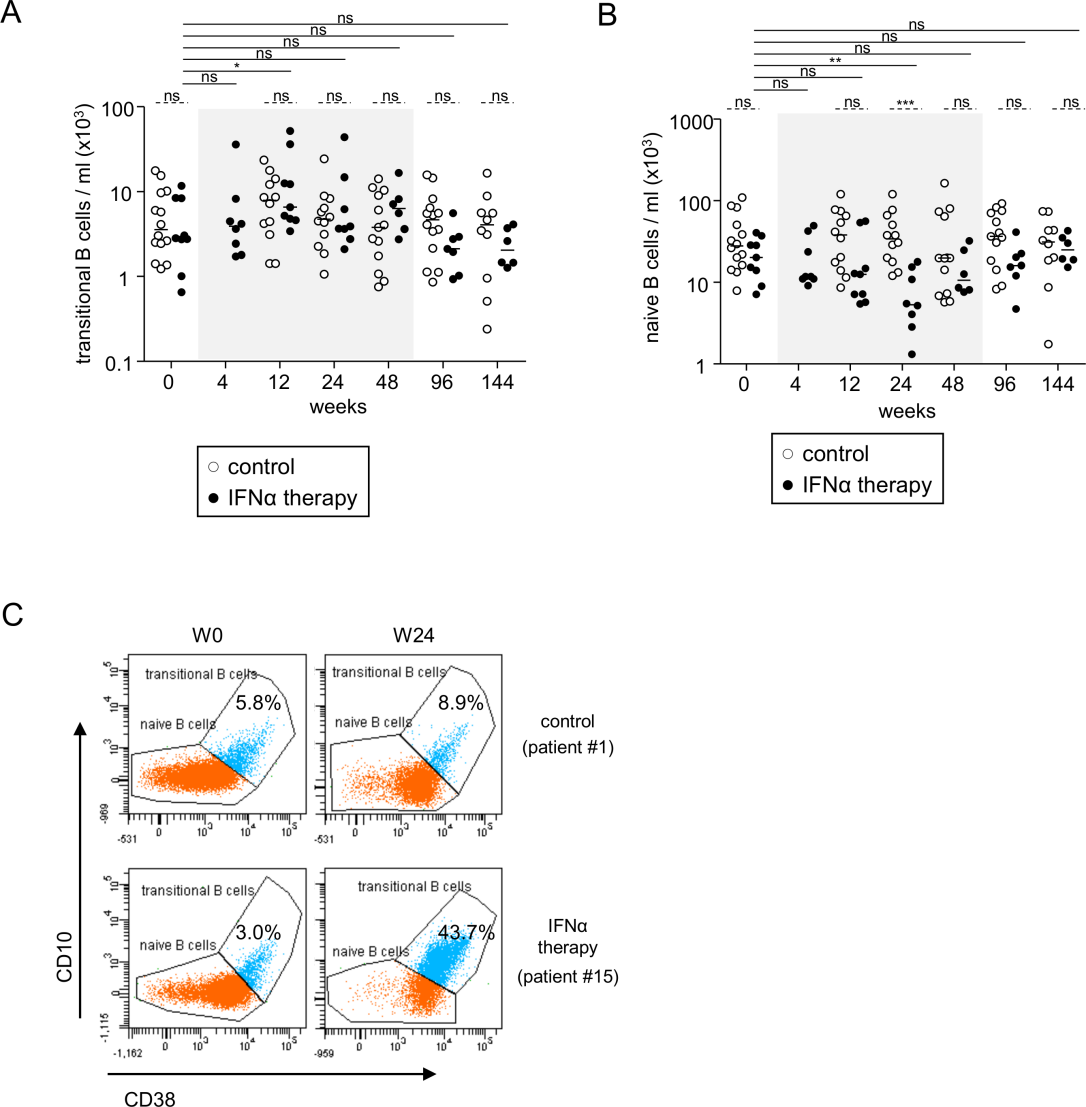
Peg-IFN-α altered peripheral transitional and naive B-cell subset distribution. Evolution of B-cell subsets was evaluated in patients with CHB before and at different time points during the treatment with nucleos(t)ide analog alone (*open circles*, n = 11–14) or together with Peg-IFN-α (*black circles*, n = 8–9). (A) Absolute numbers of transitional B-cell subsets. (B) Absolute numbers of naive B-cell subsets. (C) Representative dot plots of the frequency of transitional and naive B cells from patients treated with nucleos(t)ide analogue alone (*upper panel*) or together with Peg-IFN-α (*lower panel*) before and after 24 weeks of treatment. Dot plots are gated on CD19^+^IgD^+^CD27^-^ cells. The gray area represents the period of Peg-IFN-α administration. Bars represent median. *P* values were calculated using the Wilcoxon test (*straight lines*) or the Mann-Whitney test (*dashed lines*). * p<0.05, ** p<0.01, *** p<0.001.

We then assessed the impact of Peg-IFN-α therapy on B-cell subsets by analyzing the alterations in their frequencies and absolute numbers in both treatment arms. We used an original multi-gating approach based on the most common lineage/differentiation markers CD19, IgD, CD27, CD10, CD38, and IgM which allowed us to define eight B-cell subsets (Fig 1A) and can be found in the peripheral blood (S1 Fig). Notably, the percentages and absolute numbers of transitional B cells (CD19^+^CD27^-^IgD^+^CD10^+^CD38^high^), that is, those cells freshly released from bone-marrow, progressively significantly increased during Peg-IFN-α therapy, with the percentages peaking at W24 and the absolute numbers at W12 (Fig 2A, S2A Fig). In contrast, the frequencies and the absolute numbers of naive B cells (CD19^+^CD27^-^IgD^+^CD10^-^CD38^low^), that is, cells that differentiate from transitional B cells, progressively decreased until W24 and then increased back up to the initial levels (Fig 2B, S2B Fig). Absolute numbers of transitional and naive B cells returned to their baseline levels between 24 and 48 weeks after the beginning of Peg-IFN-α therapy. Fig 2C illustrates these striking effects on transitional and naive B cells induced by Peg-IFN-α.

**Fig 3:**
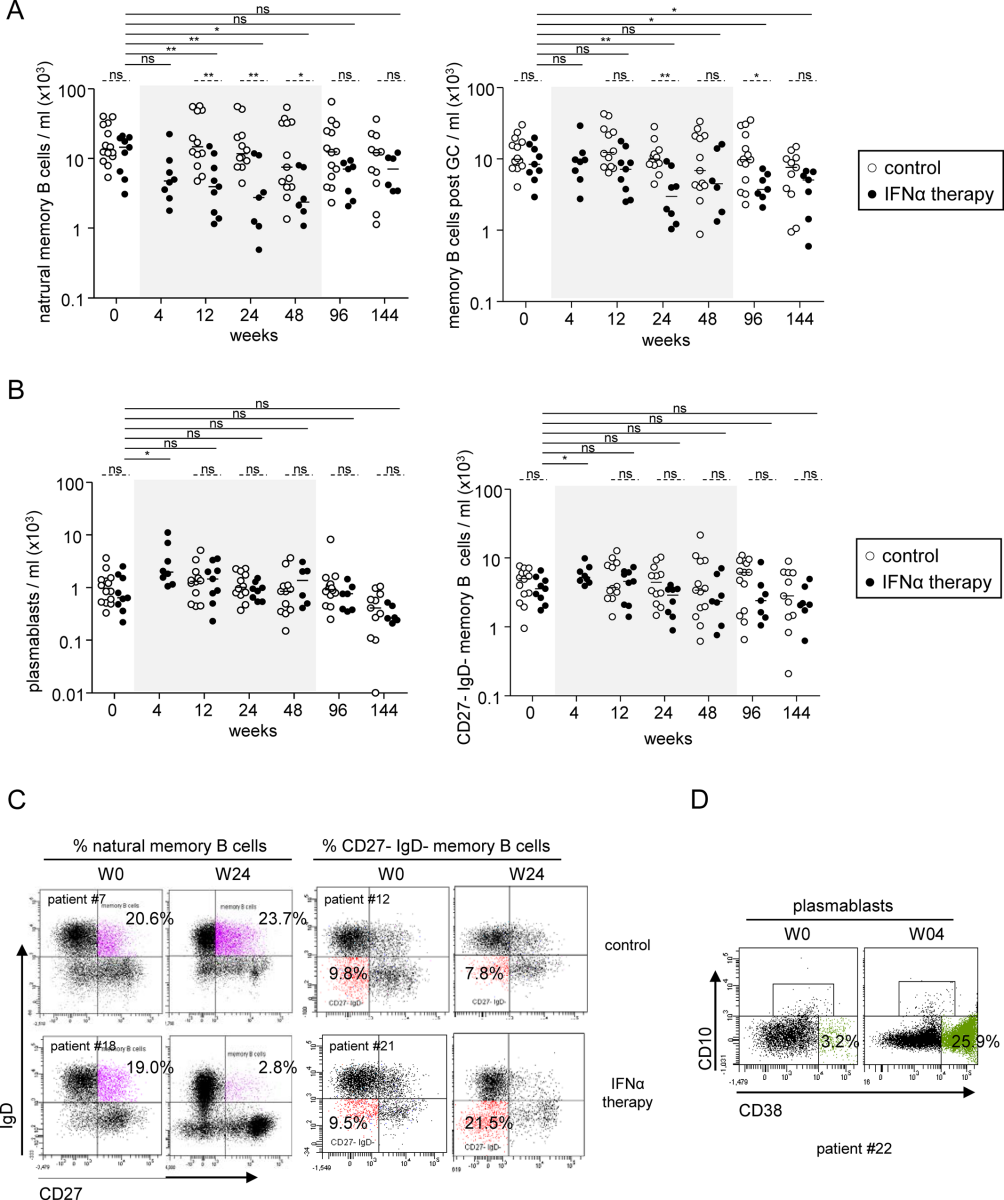
Impact of Peg-IFN-α on peripheral memory B-cell subsets and plasmablast distribution. Evolution of B cell subsets was evaluated in patients with CHB before and at different time points during the treatment with nucleos(t)ide analog alone (*open circles*, n = 11–14) or together with Peg-IFN-α (*black circles*, n = 8–9). Absolute numbers of (A) natural memory B-cell subsets (left panel), and post-GC memory B cells (right panel), (B) plasmablasts (left panel) and CD27^-^ IgD^-^ memory B-cell subsets (right panel). (C) Representative dot plots of the frequency of natural memory (left) and CD27^-^ IgD^-^ memory (right) B cells from patients treated with nucleos(t)ide analog alone (*upper panel*) or together with Peg-IFN-α (*lower panel*) before and after 24 weeks of treatment. Dot plots are gated on CD19^+^ cells. (D) Accumulation of plasmablasts during Peg-IFN-α therapy. Representative dot plots of the frequency of plasmablasts from patients treated with nucleos(t)ide analog together with Peg-IFN-α before (*left panel*) and after (*right panel*) 4 weeks of treatment. Dotplots are gated on CD19^+^IgD^-^CD27^+^ cells. The gray area represents the period of Peg-IFN-α administration. Bars represent median. *P* values were calculated using the Wilcoxon test (*straight lines*) or the Mann-Whitney test (*dashed lines*). * p<0.05, ** p<0.01, *** p<0.001.

The percentages and absolute numbers of the natural memory B cells (CD19^+^CD27^+^IgD^+^) and post-GC memory B cells (CD19^+^CD27^+^IgD^-^CD10^-^CD38^low^) also continuously decreased in response to Peg-IFN-α (Fig 3A and 3C, S2C Fig) starting as early as 4 weeks after the beginning of the treatment for the natural memory population. In contrast, we observed a significant increase in the frequency of the CD19^+^CD27^-^IgD^-^ memory B cells and plasmablasts (CD19^+^CD27^+^IgD^-^CD10^-^ CD38^high^) as early as W4 post treatment (Fig 3B, 3C and 3D, S2D Fig). These parameters were restored at the end of Peg-IFN-α treatment, except for post-GC memory B cells, whose levels were still significantly lower compared to their baseline levels more than 2 years after the cessation of Peg-IFNα therapy. Thus, Peg-IFN-α therapy mediates a complete remodeling of the B-cell compartmentalization that results in a sustained and prolonged decrease in circulating post-GC memory B cells.

We did not observe any significant changes in the global total IgG and IgM levels in the plasma of patients before or during Peg-IFN-α treatment (S3 Fig). Moreover, none of the patients developed anti-HBs antibodies during Peg-IFN-α therapy or up to 2 years after the cessation of treatment (Table 1). This suggests that changes observed in B-cell subset distribution (summarized on S4 Fig) are not related to the development of HBV-specific antibodies and do not lead to HBsAg seroconversion.

### Upregulation of sCD30 following Peg-IFN-α therapy

**Fig 4:**
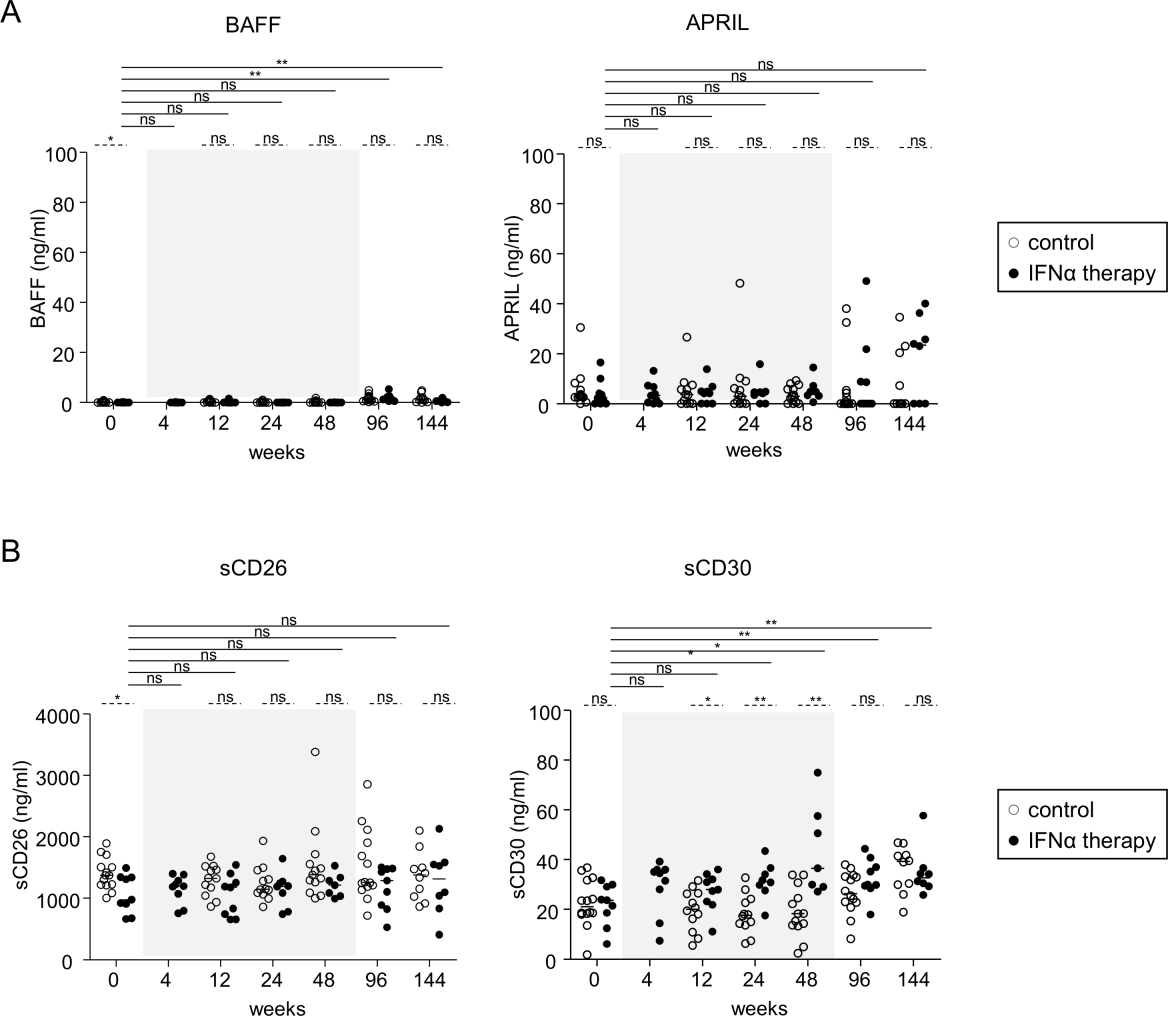
Modulation of B-cell activation signals during Peg-IFN-α therapy. Patients with CHB infection were treated with nucleos(t)ide analog alone (*open circles*, n = 11–14) or together with Peg-IFN-α (*black circles*, n = 7–9). (A,B) Plasma levels of BAFF and APRIL. (C,D) Plasma levels of soluble CD26 and soluble CD30. The gray area represents the period of Peg-IFN-α administration. Bars represent median. *P* values were calculated using the Wilcoxon test (*straight lines*) or the Mann-Whitney test (*dashed lines*). * p<0.05, ** p<0.01, *** p<0.001.

**Fig 5:**
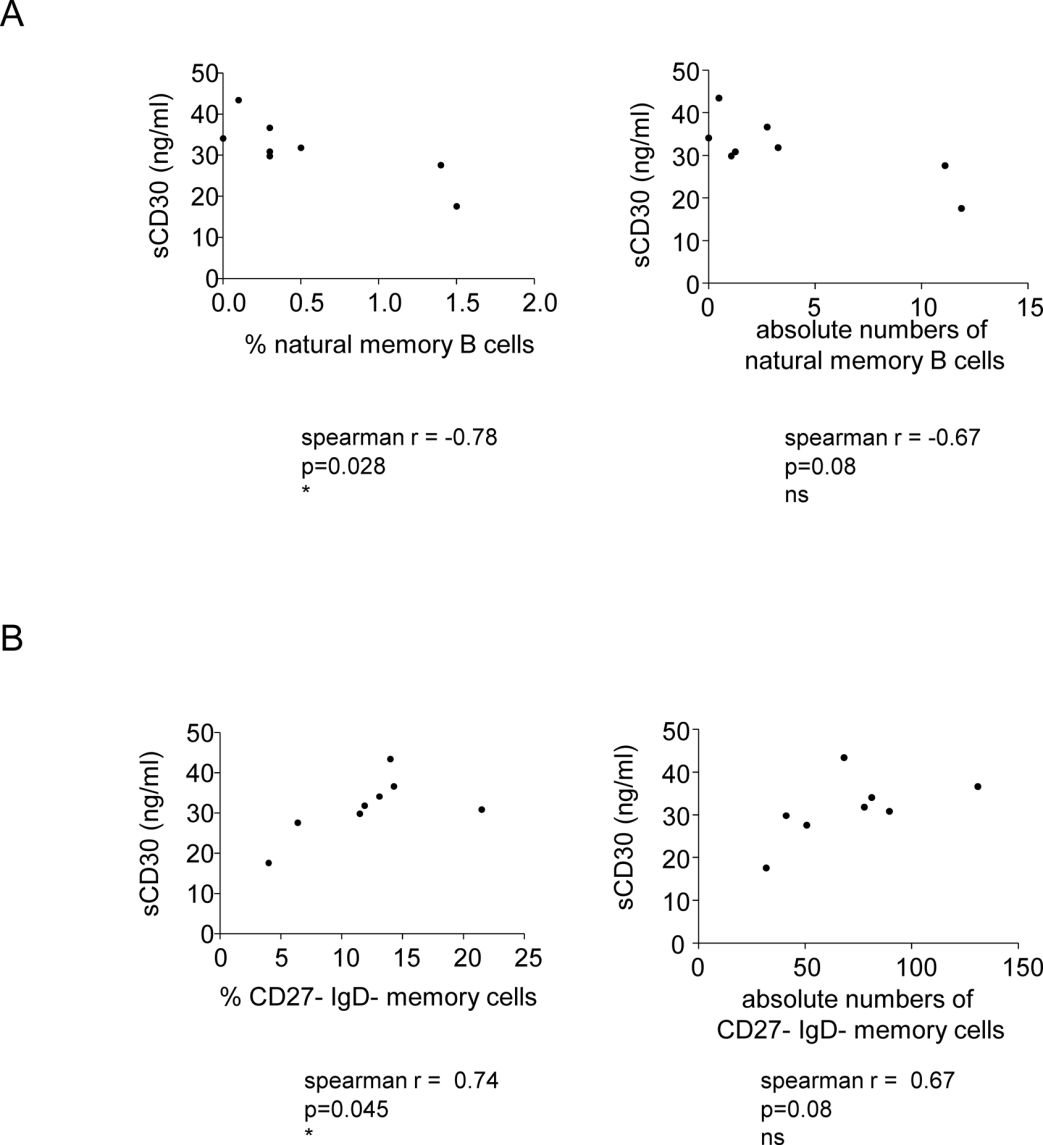
Correlations between immune modulations following Peg-IFN-α therapy. Patients with CHB infection were treated with nucleos(t)ide analog together with Peg-IFN-α (n = 8). (A) Correlations between plasma sCD30 and the percentage (left panel) and absolute numbers (right panel) of natural memory B cells at W24 of treatment. (B) Correlations between plasma sCD30 and the percentage (left panel) and absolute numbers (right panel) of CD27-IgD- memory B cells at W24 of treatment. Spearman correlation.

Because B-cell immunobiology could be modulated by BAFF, APRIL (which derived from DCs, monocytes or T cells), sCD26 and sCD30 (T-cell-derived factors), we investigated how IFN-α therapy changed their levels in the plasma of treated patients. In response to treatment, no significant changes in levels of BAFF, APRIL, or sCD26 were observed (Fig 4A and 4B). In contrast, a significant continuous increase in sCD30 levels was observed starting from W12 of Peg-IFN-α treatment initiation and peaking at W48 (from 21.99 pg/ml at W0 to 43.68 pg/ml at W48) (Fig 4B). Strikingly, even though the levels of sCD30 decreased afterwards, this modulation was still significant long (W96) after the cessation of Peg-IFN-α therapy. Interestingly, when assessing the link between sCD30 level and proportion of B-cell subsets, the sCD30 level was positively correlated with the proportion of CD27^-^IgD^-^ B cells, and negatively correlated with the proportion of natural memory B cells (Fig 5). Thus, Peg-IFN-α drastically affected the B-cell-modulating factor sCD30, which in turn could affect the repartition and activation of B-cell subsets.

### Correlation between B-cell subsets and clinical features during Peg-IFN-α therapy

**Fig 6:**
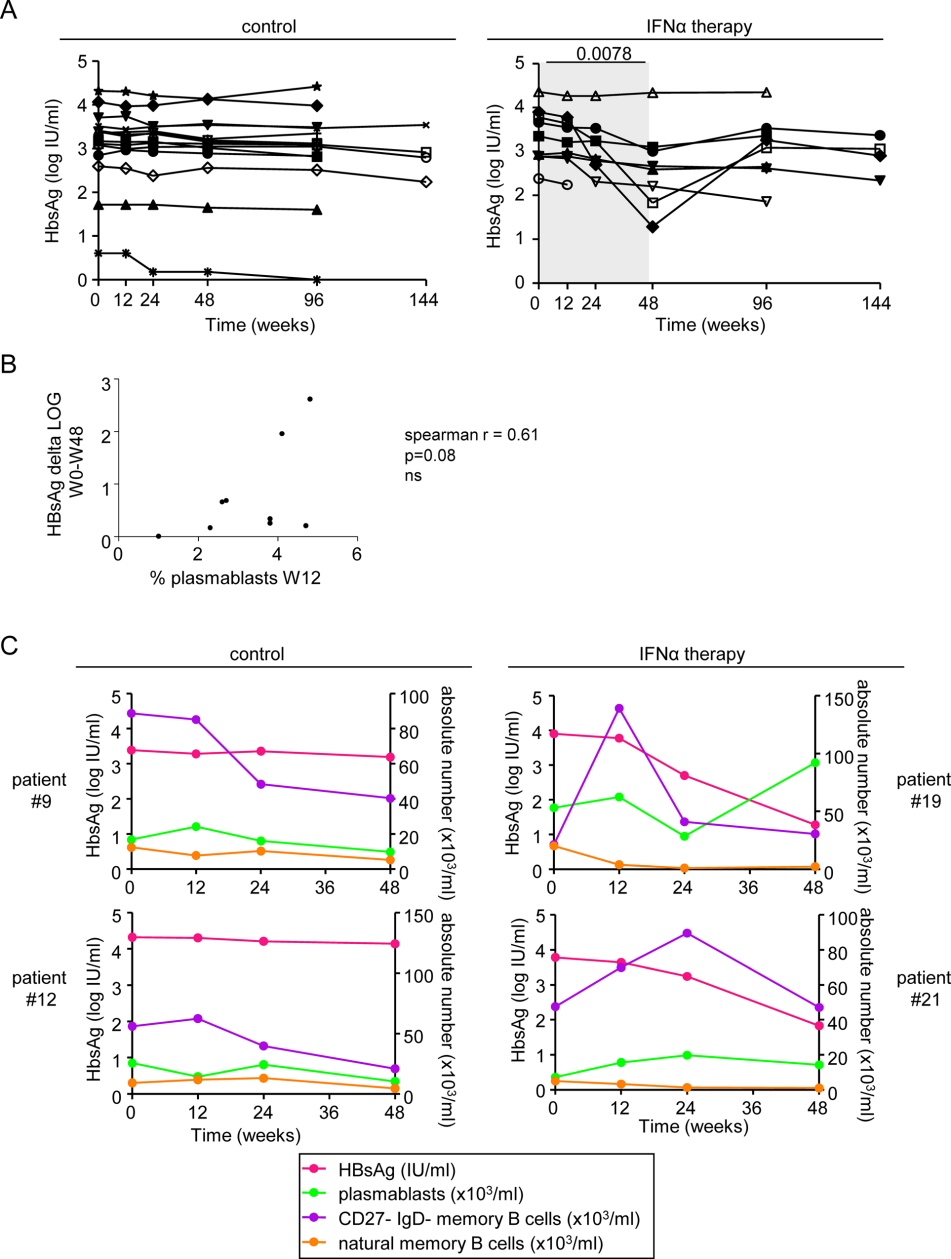
Correlations between the clinical features of patients and immune parameters. (A) Evolution of HBsAg in patients with CHB infection treated with nucleos(t)ide analog alone (*left panel*, n = 14) or together with Peg-IFN-α (*right panel*, n = 9). The gray area represents the period of Peg-IFN-α administration. (B) Correlations between the decline of HBsAg during Peg-IFN-α therapy and proportions of plasmablasts at W12. Spearman correlation. (C) Superposition of the evolution of HBsAg level and absolute numbers of B-cell subsets for patients #9 and #12 treated with NA alone, and for patients #19 and #21 treated with NA together with Peg-IFN-α.

One of the major signatures of clinical response to Peg-IFN-α therapy is the decrease in plasma HBsAg level. In contrast to the control group whose HBsAg level stayed stable throughout the study duration, this parameter significantly decreased in the group of patients receiving the additional Peg-IFN-α therapy at W48 compared to baseline, even though without reaching clearance (Fig 6A). To determine whether there is a link between changes in B-cell subset proportions and HBsAg decline, we examined correlations between these immunological and clinical parameters. Strikingly, we found a tendency for positive correlation between the elevated proportion of plasmablasts observed at W12 post-treatment and the decline in HBsAg level from baseline to W48 (Fig 6B). No other correlations between the viral parameters and the other immunologic modulations could be noticed. Two patients (#19 and #21) displayed a decline in HBsAg of more than 2 Log between baseline and W48 (Fig 6A). These patients were amongst the ones with the lowest absolute number of naive B cells, natural memory B cells, and post-GC memory B cells at W24 and W48, and the highest proportion of CD27-IgD- memory B cells at W12 or W24, suggesting a relationship between the immunologic changes observed and decline in HBsAg. The relation between changes in HBsAg and immunologic parameters are lightened up on Fig 6C. Thus, the observed changes in B-cell subsets induced by Peg-IFN-α therapy might converge towards a decline in HBsAg level.

## Discussion

Despite the critical role of B-cell responses in the control of CHB infection, the effects of Peg-IFN-α therapy on B cells have not yet been studied. Our study reveals for the first time that B-cell subsets are completely remodelled during Peg-IFN-α therapy combined with NA in patients with CHB infection, whose clinical outcomes are known to be different. It also outlines the absence of prognostic relevance of these immune modulations, as no seroconversion occurred in our cohort of patients.

Limited data are available regarding B-cell dysfunctions in patients with CHB and the impact of available therapeutic strategies on the B-cell compartment. In a previous report, a B-cell activation profile with an enhanced differentiation capacity into immunoglobulin-producing cells was observed in the context of HBV infection [15]. The lack of appropriate seroconversion paralleling the evolution towards chronicity nevertheless demonstrates a dramatic impairment in B-cell responses. Interestingly, disorders in B-cell subset phenotype and function have been described in other chronic viral infections such as in HIV-1 [16, 17] and HCV [15, 18, 19]. The mechanisms leading to these dysfunctions as well as the impact of the current therapies on the B-cell population have not been elucidated in chronic viral infection. In this study, we reveal that Peg-IFN-α over NA treatment may reverse the dysfunctions of B cells induced by HBV.

We observed a progressive decrease in peripheral B-cell count following Peg-IFN-α treatment, which fits with the drastic reduction in circulating T lymphocytes reported during the course of Peg-IFN-α therapy [5]. We also found major changes in the repartition of B-cell subsets, suggesting that Peg-IFN-α has a potent effect on B-cell responses. Indeed, the transitional B cells and plasmablasts continuously increased during Peg-IFN-α therapy. Interestingly, transitional B cells bridge primary B-cell development in bone marrow and B-cell maturation in lymphoid organs. Their increase in the periphery may reflect a higher rate of B-cell generation and differentiation towards plasmablasts or CD27^-^ memory B cells, and an elevation of immunoglobulin-secreting B cells that may ultimately favor HBsAg seroconversion. Noteworthy, such modulation tends to correlate with the decrease in plasma HBsAg, directly linking B-cell modulation with virological parameters. As plasmablasts are precursors of plasma cells, it might means that Peg-IFN-α therapy may favor the development of anti-HBV immunity, leading to anti-HBs seroconversion and elimination of HBsAg. Yet none of the studied patients achieved seroconversion in our study. The two patients with the highest HBsAg rate decrease didn’t seroconvert but their HBsAg rate increase back to the baseline level after the stop of Peg-IFN-α treatment. This questions whether the duration of Peg-IFN-α therapy is optimal or not to favour seroconversion. The mechanisms developed by HBV to escape immunity may directly or indirectly prevent the successful differentiation and maturation of B-cell plasmablasts triggered by Peg-IFN-α, impairing the elimination of infection. Indeed, it is known that HBV inhibits TLR9-mediated pathways [20], which may be required to successfully drive specific B-cell responses [21]. It has been reported that patients with systemic lupus erythematosus, who are characterized by elevated type-I IFN levels, have a higher representation of peripheral transitional B cells [22], together with higher numbers of plasmablasts [23] and an expanded population of CD27^-^IgD^-^ memory B-cell subset [24]. The CD27^-^IgD^-^ memory B cells represent an intermediate step in the differentiation into plasma cells. All these features were also found in our study of patients with CHB during the course of Peg-IFN-α therapy, which supports the major role of type-I IFN in modulating B-cell subsets. In addition to its direct contribution to B-cell differentiation and antibody production [25], type-I IFN may be critical for the long-lasting sequestration of B cells within the follicles [26], which may subsequently favor antigen delivery to DCs and interactions between T and B cells of same specificity [27]. IFN-α also modulates many cell subsets, such as DCs and NK cells, that in turn may affect B cells [28, 29]. These multiple effects of type-I IFN may together modulate B-cell repartition and function through different cellular pathways. The modulations observed in peripheral B-cell distribution could also result from an altered homing of specific subsets or a re-compartmentalization of the B-cell subtypes. In light of our data, it would be interesting to further study the global B-cell subset distribution, also hardly evident in humans.

Interestingly, we found a sustained increase in sCD30 levels triggered by Peg-IFN-α, which correlated with the changes in B-cell subsets. The CD30/CD30L pathway plays a major role in immunological processes, especially in the regulation of humoral immune responses [30]. High serum levels of sCD30 have been reported to correlate with disease activity in several autoimmune diseases, which are driven by type-I IFN [30]. High levels of sCD30 are also found during acute HBV infection, where it participates in the development of immunity [31] as well as during the active phase of chronic hepatitis [32]. Because sCD30 is released by activated T cells, and B cells can be activated by T-helper cells [15], our observations suggest that Peg-IFN-α may trigger a T-cell-dependent B-cell remodeling. This hypothesis is sustained by the observation that patients receiving Peg-IFN-α therapy had enhanced HBV-specific T-cell responses [33, 34] (and personal data on the same cohort of patients, not shown). Strikingly, we found a correlation between sCD30 level and the proportion of CD27^-^IgD^-^ memory B cells. This suggests that the modulation of T cells by Peg-IFN-α potentially triggers the release of sCD30, which subsequently may affect B-cell subsets. In addition, T-follicular helper cells secrete cytokines that will favour B-cell proliferation and differentiation within the germinal centers, and will sustain GC reactions, which will trigger the emergence of antibody-secreting plasma cells and memory B cells [35].

Despite the observed immunologic modulations, none of the patients achieved HBsAg seroconversion following Peg-IFN-α therapy. However, our study highlighted a trend towards a positive relationship between increased proportions and numbers of plasmablasts and decreased plasma HBsAg loads in some patients. Even though the changes in the B-cell compartment did not persist after ending treatment, our observations suggest that the immunologic changes triggered by Peg-IFN-α therapy can positively affect viral parameters. Overall, Peg-IFN-α may trigger modulations of the B-cell compartment either directly by influencing B-cell differentiation and antibody production [25] or indirectly by activating other immune cells such as dendritic cells and T cells that can subsequently modulate B-cell differentiation and function [36].

Our pilot study revealed that the potent immunomodulatory properties of Peg-IFN-α mediates a complete remodeling of B-cell compartmentalization and provides new insights into the immunomodulatory effects of Peg-IFN-α on B cells. However, as shown for other parameters[5, 37, 38], all the immune changes returned to baseline after the cessation of Peg-IFN-α treatment. The observed immune alterations being not prognostically relevant, our work also questioned the benefit of the add-on Peg-IFN-α treatment over the NA or Peg-IFN-α monotherapies.

## Supporting Information

S1 Fig Overview of the differentiation stages of B cells. The figure shows the dynamic of the eight B-cell subsets as defined by CD19, CD10, CD27, CD38, IgD, and IgM markers found in the peripheral blood.
(TIFF)

S2 Fig Peg-IFN-α altered peripheral transitional and naive B-cell subset distribution and impacted peripheral memory B-cell subsets and plasmablasts distribution. Evolution of B-cell subsets was evaluated in patients with CHB before and at different time points during the treatment with nucleos(t)ide analog alone (open circles, n = 11–14) or together with Peg-IFN-α (black circles, n = 8–9). (A) Frequencies of transitional B-cell subsets. (B) Frequencies of naive B-cell subsets. (C) Frequencies of natural memory B-cell subsets (left panel) and post-GC memory B cells (right panel). (D) Frequencies of plasmablasts (left panel) and CD27- IgD- memory B-cell subsets (right panel). The gray area represents the period of Peg-IFN-α administration. Bars represent median. P values were calculated using the Wilcoxon test (straight lines) or the Mann-Whitney test (dashed lines). * p<0.05, ** p<0.01, *** p<0.001.
(TIFF)

S3 Fig Quantification of total immunoglobulins during the course of Peg-IFN-α therapy. Plasma levels of IgG (left) and IgM (right) from patients with CHB infection treated with nucleos(t)ide analog alone (open circles, n = 12–14) or together with Peg-IFN-α (black circles, n = 7–9). The gray area represents the period of Peg-IFN-α administration. Bars represent median. P values were calculated using the Wilcoxon test (straight lines) or the Mann-Whitney test (dashed lines).
(TIFF)

S4 Fig Overview of B-cell subset distribution during Peg-IFN-α therapy. Distribution of the major circulating B-cell subsets was evaluated in CHB patients before and at different time points during the treatment with nucleos(t)ide analog alone (upper panel, n = 11–14) or together with Peg-IFN-α (bottom panel, n = 8–9). The gray area represents the period of Peg-IFN-α administration.
(TIFF)

## References

[1] Lampertico P, Vigano M, Di Costanzo GG, Sagnelli E, Fasano M, Di Marco V, et al Randomised study comparing 48 and 96 weeks peginterferon alpha-2a therapy in genotype D HBeAg-negative chronic hepatitis B. Gut. 2013;62(2):290–8. Epub 2012/08/04. 10.1136/gutjnl-2011-301430 . 22859496

[2] Marcellin P, Bonino F, Lau GK, Farci P, Yurdaydin C, Piratvisuth T, et al Sustained response of hepatitis B e antigen-negative patients 3 years after treatment with peginterferon alpha-2a. Gastroenterology. 2009;136(7):2169–79 e1-4. Epub 2009/03/24. 10.1053/j.gastro.2009.03.006 . 19303414

[3] Rijckborst V, ter Borg MJ, Cakaloglu Y, Ferenci P, Tabak F, Akdogan M, et al A randomized trial of peginterferon alpha-2a with or without ribavirin for HBeAg-negative chronic hepatitis B. Am J Gastroenterol. 2010;105(8):1762–9. Epub 2010/05/13. 10.1038/ajg.2010.186 . 20461068

[4] EASL clinical practice guidelines: Management of chronic hepatitis B virus infection. Journal of hepatology. 2012;57(1):167–85. Epub 2012/03/23. 10.1016/j.jhep.2012.02.010 . 22436845

[5] Micco L, Peppa D, Loggi E, Schurich A, Jefferson L, Cursaro C, et al Differential boosting of innate and adaptive antiviral responses during pegylated-interferon-alpha therapy of chronic hepatitis B. Journal of hepatology. 2013;58(2):225–33. Epub 2012/10/11. 10.1016/j.jhep.2012.09.029 . 23046671

[6] Rehermann B, Lau D, Hoofnagle JH, Chisari FV. Cytotoxic T lymphocyte responsiveness after resolution of chronic hepatitis B virus infection. The Journal of clinical investigation. 1996;97(7):1655–65. Epub 1996/04/01. 10.1172/JCI118592 8601631 PMC507230

[7] Liu YZ, Hou FQ, Ding P, Ren YY, Li SH, Wang GQ. Pegylated interferon alpha enhances recovery of memory T cells in e antigen positive chronic hepatitis B patients. Virol J. 2012;9:274 Epub 2012/11/20. 10.1186/1743-422X-9-274 23158844 PMC3518195

[8] Stelma F, de Niet A, Tempelmans Plat-Sinnige MJ, Jansen L, Takkenberg RB, Reesink HW, et al Natural Killer Cell Characteristics in Patients With Chronic Hepatitis B Virus (HBV) Infection Are Associated With HBV Surface Antigen Clearance After Combination Treatment With Pegylated Interferon Alfa-2a and Adefovir. The Journal of infectious diseases. 2015 Epub 2015/03/21. 10.1093/infdis/jiv180 . 25791117

[9] Chisari FV, Isogawa M, Wieland SF. Pathogenesis of hepatitis B virus infection. Pathol Biol (Paris). 2010;58(4):258–66. Epub 2010/02/02. 10.1016/j.patbio.2009.11.001 20116937 PMC2888709

[10] Rehermann B, Nascimbeni M. Immunology of hepatitis B virus and hepatitis C virus infection. Nature reviews Immunology. 2005;5(3):215–29. Epub 2005/03/02. 10.1038/nri1573 . 15738952

[11] Garraud O, Borhis G, Badr G, Degrelle S, Pozzetto B, Cognasse F, et al Revisiting the B-cell compartment in mouse and humans: more than one B-cell subset exists in the marginal zone and beyond. BMC immunology. 2012;13:63 Epub 2012/12/01. 10.1186/1471-2172-13-63 23194300 PMC3526508

[12] LeBien TW, Tedder TF. B lymphocytes: how they develop and function. Blood. 2008;112(5):1570–80. Epub 2008/08/30. 10.1182/blood-2008-02-078071 18725575 PMC2518873

[13] Jackson SM, Wilson PC, James JA, Capra JD. Human B cell subsets. Advances in immunology. 2008;98:151–224. Epub 2008/09/06. 10.1016/S0065-2776(08)00405-7 . 18772006

[14] Sanz I, Wei C, Lee FE, Anolik J. Phenotypic and functional heterogeneity of human memory B cells. Seminars in immunology. 2008;20(1):67–82. Epub 2008/02/09. 10.1016/j.smim.2007.12.006 18258454 PMC2440717

[15] Oliviero B, Cerino A, Varchetta S, Paudice E, Pai S, Ludovisi S, et al Enhanced B-cell differentiation and reduced proliferative capacity in chronic hepatitis C and chronic hepatitis B virus infections. Journal of hepatology. 2011;55(1):53–60. Epub 2010/12/15. 10.1016/j.jhep.2010.10.016 . 21145853

[16] Amu S, Ruffin N, Rethi B, Chiodi F. Impairment of B-cell functions during HIV-1 infection. AIDS. 2013;27(15):2323–34. Epub 2013/04/19. 10.1097/QAD.0b013e328361a427 . 23595152

[17] Pensieroso S, Galli L, Nozza S, Ruffin N, Castagna A, Tambussi G, et al B-cell subset alterations and correlated factors in HIV-1 infection. AIDS. 2013;27(8):1209–17. Epub 2013/01/25. 10.1097/QAD.0b013e32835edc47 . 23343911

[18] Ni J, Hembrador E, Di Bisceglie AM, Jacobson IM, Talal AH, Butera D, et al Accumulation of B lymphocytes with a naive, resting phenotype in a subset of hepatitis C patients. J Immunol. 2003;170(6):3429–39. Epub 2003/03/11. . 12626604 10.4049/jimmunol.170.6.3429

[19] Racanelli V, Frassanito MA, Leone P, Galiano M, De Re V, Silvestris F, et al Antibody production and in vitro behavior of CD27-defined B-cell subsets: persistent hepatitis C virus infection changes the rules. Journal of virology. 2006;80(8):3923–34. Epub 2006/03/31. 10.1128/JVI.80.8.3923-3934.2006 16571809 PMC1440441

[20] Vincent IE, Zannetti C, Lucifora J, Norder H, Protzer U, Hainaut P, et al Hepatitis B virus impairs TLR9 expression and function in plasmacytoid dendritic cells. PloS one. 2011;6(10):e26315 Epub 2011/11/03. 10.1371/journal.pone.0026315 22046272 PMC3201951

[21] Fathallah I, Parroche P, Gruffat H, Zannetti C, Johansson H, Yue J, et al EBV latent membrane protein 1 is a negative regulator of TLR9. J Immunol. 2010;185(11):6439–47. Epub 2010/10/29. 10.4049/jimmunol.0903459 . 20980631

[22] Sims GP, Ettinger R, Shirota Y, Yarboro CH, Illei GG, Lipsky PE. Identification and characterization of circulating human transitional B cells. Blood. 2005;105(11):4390–8. Epub 2005/02/11. 10.1182/blood-2004-11-4284 15701725 PMC1895038

[23] Wehr C, Eibel H, Masilamani M, Illges H, Schlesier M, Peter HH, et al A new CD21low B cell population in the peripheral blood of patients with SLE. Clin Immunol. 2004;113(2):161–71. Epub 2004/09/29. 10.1016/j.clim.2004.05.010 . 15451473

[24] Wei C, Anolik J, Cappione A, Zheng B, Pugh-Bernard A, Brooks J, et al A new population of cells lacking expression of CD27 represents a notable component of the B cell memory compartment in systemic lupus erythematosus. J Immunol. 2007;178(10):6624–33. Epub 2007/05/04. . 17475894 10.4049/jimmunol.178.10.6624

[25] Jego G, Palucka AK, Blanck JP, Chalouni C, Pascual V, Banchereau J. Plasmacytoid dendritic cells induce plasma cell differentiation through type I interferon and interleukin 6. Immunity. 2003;19(2):225–34. Epub 2003/08/23. . 12932356 10.1016/s1074-7613(03)00208-5

[26] Shiow LR, Rosen DB, Brdickova N, Xu Y, An J, Lanier LL, et al CD69 acts downstream of interferon-alpha/beta to inhibit S1P1 and lymphocyte egress from lymphoid organs. Nature. 2006;440(7083):540–4. Epub 2006/03/10. 10.1038/nature04606 . 16525420

[27] Cinamon G, Zachariah MA, Lam OM, Foss FW Jr, Cyster JG. Follicular shuttling of marginal zone B cells facilitates antigen transport. Nature immunology. 2008;9(1):54–62. Epub 2007/11/27. 10.1038/ni1542 18037889 PMC2488964

[28] Swiecki M, Colonna M. Type I interferons: diversity of sources, production pathways and effects on immune responses. Curr Opin Virol. 2011;1(6):463–75. Epub 2012/03/24. 10.1016/j.coviro.2011.10.026 22440910 PMC3572907

[29] Gonzalez-Navajas JM, Lee J, David M, Raz E. Immunomodulatory functions of type I interferons. Nature reviews Immunology. 2012;12(2):125–35. Epub 2012/01/10. 10.1038/nri3133 . 22222875 PMC3727154

[30] Kennedy MK, Willis CR, Armitage RJ. Deciphering CD30 ligand biology and its role in humoral immunity. Immunology. 2006;118(2):143–52. Epub 2006/06/15. 10.1111/j.1365-2567.2006.02354.x 16771849 PMC1782289

[31] Monsalve-De Castillo F, Romero TA, Estevez J, Costa LL, Atencio R, Porto L, et al Concentrations of cytokines, soluble interleukin-2 receptor, and soluble CD30 in sera of patients with hepatitis B virus infection during acute and convalescent phases. Clinical and diagnostic laboratory immunology. 2002;9(6):1372–5. Epub 2002/11/05. 12414777 10.1128/CDLI.9.6.1372-1375.2002 PMC130099

[32] Fattovich G, Vinante F, Giustina G, Morosato L, Alberti A, Ruol A, et al Serum levels of soluble CD30 in chronic hepatitis B virus infection. Clinical and experimental immunology. 1996;103(1):105–10. Epub 1996/01/01. 8565268 10.1046/j.1365-2249.1996.915607.x PMC2200324

[33] Sprinzl MF, Russo C, Kittner J, Allgayer S, Grambihler A, Bartsch B, et al Hepatitis B virus-specific T-cell responses during IFN administration in a small cohort of chronic hepatitis B patients under nucleos(t)ide analogue treatment. Journal of viral hepatitis. 2014;21(9):633–41. Epub 2013/11/21. 10.1111/jvh.12189 . 24251783

[34] Thimme R, Dandri M. Dissecting the divergent effects of interferon-alpha on immune cells: time to rethink combination therapy in chronic hepatitis B? Journal of hepatology. 2013;58(2):205–9. Epub 2012/11/20. 10.1016/j.jhep.2012.11.007 . 23159772

[35] Shlomchik MJ, Weisel F. Germinal center selection and the development of memory B and plasma cells. Immunological reviews. 2012;247(1):52–63. Epub 2012/04/17. 10.1111/j.1600-065X.2012.01124.x . 22500831

[36] Garcia-Sastre A, Biron CA. Type 1 interferons and the virus-host relationship: a lesson in detente. Science. 2006;312(5775):879–82. Epub 2006/05/13. 10.1126/science.1125676 . 16690858

[37] Boltjes A, Op den Brouw ML, Biesta PJ, Binda RS, van der Molen RG, Boonstra A, et al Assessment of the effect of ribavirin on myeloid and plasmacytoid dendritic cells during interferon-based therapy of chronic hepatitis B patients. Mol Immunol. 2013;53(1–2):72–8. Epub 2012/07/21. 10.1016/j.molimm.2012.06.016 . 22814486

[38] Penna A, Laccabue D, Libri I, Giuberti T, Schivazappa S, Alfieri A, et al Peginterferon-alpha does not improve early peripheral blood HBV-specific T-cell responses in HBeAg-negative chronic hepatitis. Journal of hepatology. 2012;56(6):1239–46. Epub 2012/02/14. 10.1016/j.jhep.2011.12.032 . 22326467

